# Feasibility study of performing IGRT system daily QA using a commercial QA device

**DOI:** 10.1120/jacmp.v12i3.3535

**Published:** 2011-06-01

**Authors:** Jean L.Peng, Darren Kahler, Jonathan G. Li, Robert J.Amdur, Kenneth N. Vanek, Chihray Liu

**Affiliations:** ^1^ Department of Radiation Oncology Medical University of South Carolina Charleston South Carolina 29425; ^2^ Department of Radiation Oncology University of Florida Health Science Center Gainesville Florida 32610‐0385 USA

**Keywords:** daily QA, multiple detectors, image‐guided stereotactic positioning system

## Abstract

The purpose of this study was to investigate the feasibility of using a single QA device for comprehensive, efficient daily QA of a linear accelerator (Linac) and three image‐guided stereotactic positioning systems (IGSPSs). The Sun Nuclear Daily QA 3 (DQA3) device was used to perform daily dosimetry and mechanical accuracy tests for an Elekta Linac, as well as daily image geometric and isocenter coincidence accuracy tests for three IGSPSs: the AlignRT surface imaging system; the frameless SonArray optical tracking System (FSA) and the Elekta kV CBCT. The DQA3 can also be used for couch positioning, repositioning, and rotational tests during the monthly QA. Based on phantom imaging, the Linac coordinate system determined using AlignRT was within 0.7 mm/0.6° of that of the CBCT system. The difference is attributable to the different calibration methods that are utilized for these two systems. The laser alignment was within 0.5 mm of the isocenter location determined with the three IGSPSs. The ODI constancy was ± 0.5 mm. For gantry and table angles of 0°, the mean isocenter displacement vectors determined using the three systems were within 0.7 mm and 0.6° of one another. Isocenter rotational offsets measured with the systems were all ≤ 0.5°. For photon and electron beams tested over a period of eight months, the output was verified to remain within 2%, energy variations were within 2%, and the symmetry and flatness were within 1%. The field size and light‐radiation coincidence were within 1mm ± 1 mm. For dosimetry reproducibility, the standard deviation was within 0.2% for all tests and all energies, except for photon energy variation which was 0.6%. The total measurement time for all tasks took less than 15 minutes per QA session compared to 40 minutes with our previous procedure, which utilized an individual QA device for each IGSPS. The DQA3 can be used for accurate and efficient Linac and IGSPS daily QA. It shortens QA device setup time, eliminates errors introduced by changing phantoms to perform different tests, and streamlines the task of performing dosimetric checks.

PACS number: 87.56.Fc

## I. INTRODUCTION

The American Association of Physicists in Medicine (AAPM) Task Group (TG)‐40 recommends that a megavoltage radiation therapy unit's output be checked every morning before treatment begins.^(1)^ Linac daily checks are a main component of any quality assurance (QA) program.

The QA procedures involve periodic measurements of specified parameters using dedicated tools that are validated with specific acceptability criteria and tolerance levels to ensure that hardware and software functions safely and reliably perform as expected. Vendors may provide instructions on how to perform the measurements and how to use the tools for clinical sites. Users establish their own measurement protocols, so variations in QA methods naturally exist between institutions. A consistent set of measurement criteria is ideal; to this end, the AAPM TG142 report, an update to the TG‐40 report, specifies new tests and tolerances.^(2)^ The updated report includes recommendations for QA parameters, as well as their measurement frequencies and acceptability criteria.

With the steep dose gradients and tight tumor margins created with intensity‐modulated radiotherapy (IMRT), potential setup errors and organ motion lead to a greater chance of increased dose to organs at risk (OARs). Image‐guided radiation therapy (IGRT), wherein imaging devices are used to ensure that the radiation delivery adheres as closely as possible to the original plan, can help to minimize these problems. Additionally, IGSPSs employ various methods to achieve accurate target localization in stereotactic radiotherapy (SRT);^(3)^ the results are summarized in the AAPM TG‐68 report.^(3)^ The use of this new technology necessitates a comprehensive QA program to monitor and maintain the system performance characteristics that were established when these systems were commissioned. QA phantoms and measurement methods may differ for different IGSPSs and institutions; however, several recent AAPM TG efforts^(^ ^2^ ^,^ ^4^ ^)^ and other studies^(^ ^5^ ^,^ ^6^ ^)^ address the QA issues associated with radiographic systems. The use of a single device for QA of both the Linac and radiographic and nonradiographic IGSPSs would greatly streamline the QA process. An additional benefit would be the elimination of setup uncertainties that are introduced by repositioning multiple QA devices and/or phantoms.

**Figure 1:**
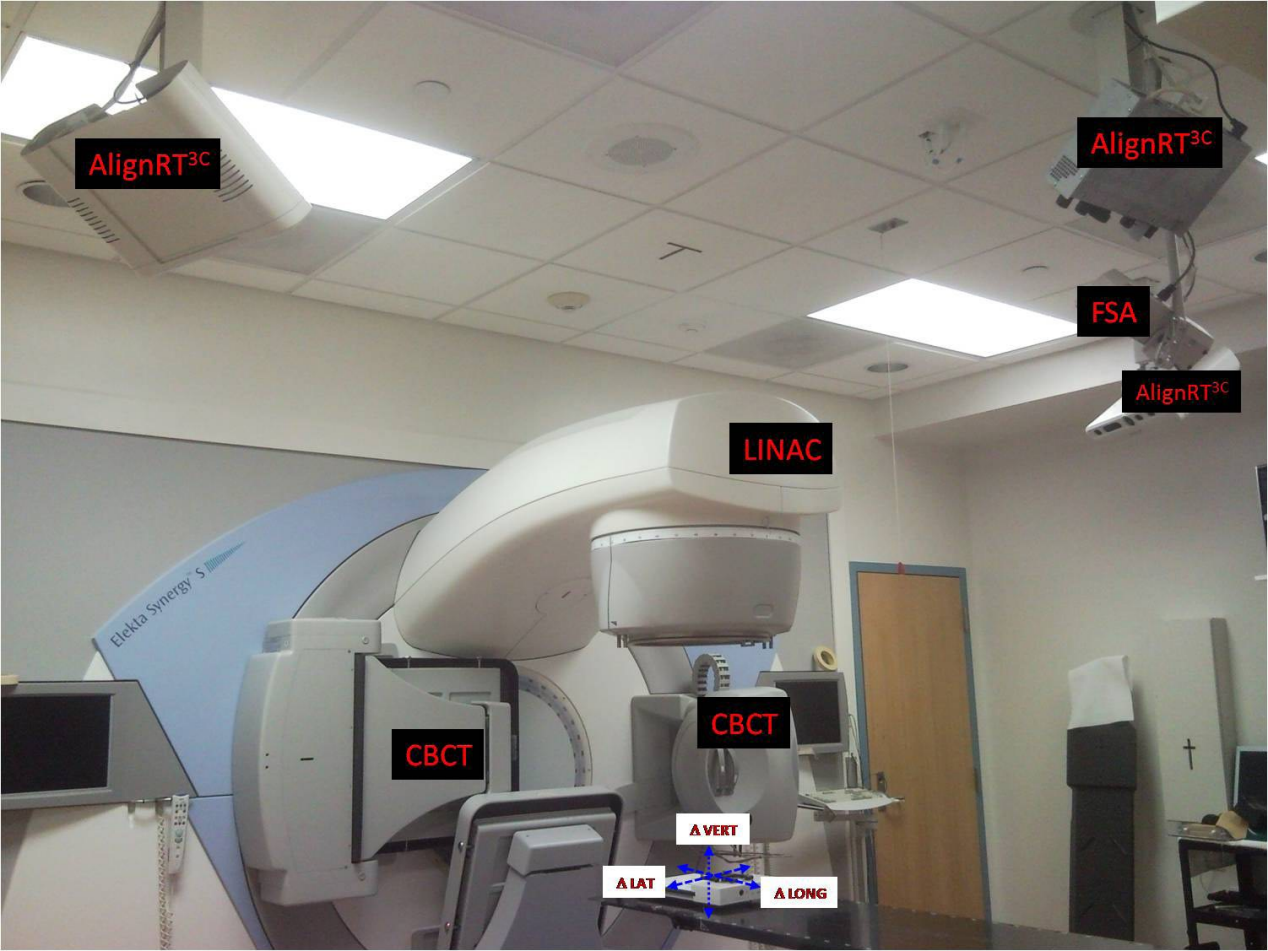
Photograph of the Linac room with three IGSPSs: CBCT, FSA, and ${\text{AlignRT}}^{3C}$. The arrows indicate the three translational and rotational directions: Δ LAT = lateral (left–right), Δ LONG = longitudinal (superior–inferior), and Δ VERT = vertical (anterior–posterior).

In a typical QA procedure, radiographic images of a phantom are obtained to verify the system's ability to correctly position objects with respect to radiation isocenter,^(^ ^5^ ^,^ ^6^ ^)^ and nonradiographic systems are limited by the accuracy of their correlation with the radiation ioscenter. ^(^ ^7^ ^,^ ^8^ ^)^ At our institution, three IGSPSs are housed together with an Elekta Linac (Elekta Oncology System, Norcross, GA): the AlignRT 3D system with three camera pods (Align ${\text{RT}}^{3C}$, Vision RT Limited, London, UK), the frameless SonArray Infrared Optical Tracking System (FSA) (Zmed/Varian, Inc, Ashland, MA), and the Elekta kilovoltage (kV) Cone‐Beam Computed Tomography System (CBCT) (X‐ray Volume Imaging, Elekta Oncology Systems, Norcross, GA), (Fig. 1). These imaging systems were calibrated per vendor instructions to match the Linac and room coordinate systems. For daily QA, the therapists perform dosimetric checks of the Linac and geometric accuracy tests of the three IGSPSs using vendor‐provided or custom‐designed in‐house phantoms. Switching phantoms between tests is inefficient and may also introduce additional setup errors. To simplify and improve the daily QA procedure, we designed a straightforward, comprehensive, daily QA program that utilizes the commercial Daily QA 3 (DQA3) device (Sun Nuclear Inc, Melbourne, FL) for both the Linac and all three of the IGSPs. The program consists of three sets of checks: dosimetry, mechanical (couch and laser), and imaging geometry. Here we describe the program and present the results of measurements that were taken over an extended period of time to explore the feasibility of its implementation for routine daily QA.

## II. MATERIALS AND METHODS

### A. Description of the Daily QA 3 device

**Figure 2:**
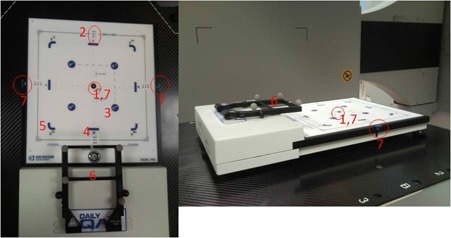
Bite frame attached to the DailyQA3 device QA phantom used for the investigation of positioning accuracy between the three IGSPSs. Notes: 1=CAX chamber, 2=field size diodes, 3=electron energy chambers, 4=field size chambers, 5=photon energy chambers, 6=bite frame, 7=radiopaque markers.

The commercially available DQA3 has been described previously.^(9)^ It consists of 25 detectors; 13 parallel plate chambers and 12 diodes within a $20\times 20\;{\text{cm}}^{2}$ detection area (Fig. 2); one chamber at the central axis (CAX); four rectangular ion chambers 8 cm from the CAX that are used for the flatness, symmetry, and output checks; four curved ionization chambers located at the corners of a 16 cm^2^ square that are used for photon energy checks; four circular chambers with inherent attenuators for electron energy verification; and four sets of three diodes with 5 mm spacing along the central axis for the radiation field size check. The device performs automatic temperature and pressure correction, and the measured data is automatically recorded in its database software (ATLAS, Sun Nuclear Inc, Melbourne, FL).

In order to eliminate differences among the individual detector readings, an array calibration must be performed under the same irradiation conditions as those of the daily QA measurements. In our study, the array was calibrated using a $20\times 20\;{\text{cm}}^{2}$ field size with a 100 cm source‐to‐surface distance (SSD) to determine the relative sensitivity of the detectors. This information was stored as a set of specific calibration factors for the detectors to be applied to the raw measurements. The above setup is used and the baseline data are acquired for all detectors at once; subsequent data is collected daily to compare with the baseline.

### B. Description of the three IGSPSs

The Align ${\text{RT}}^{3C}$ system^(7)^ was designed to acquire 3D surface images using one central and two side camera pods; several stereoscopically‐arranged charge‐coupled device (CCD) cameras compute the surface images and provide gray‐level and dynamic images. All images are reconstructed as a three‐dimensional surface composed of 10,000 points spaced approximately 1 to 3 mm apart. The Align ${\text{RT}}^{3C}$ system supports static and continuous real‐time surface image displays. All measurements were performed in real‐time mode, for which the surface image capture frame rate is approximately 1.5 frames per second. Additionally, the software (version 4.2) supports two types of surface image references: skin contours reconstructed from planning CT images, and previously recorded optical surface images. The version 4.3 software offers 3D surface image fusion in real time with a user‐defined ROI selection. Camera calibration is performed using the light field and room lasers on a daily and monthly basis, as recommended by the manufacturer. The calibration is reproducible to within 0.8 mm.^(7)^

The FSA system is a 2‐CCD infra‐red camera system.^(5)^ A custom bite plate with two nonreflective and four passive–reflective spherical markers is affixed to a patient's maxillary dentition to form a rigid system. The patient's position, which is determined by registering the reflective marker positions with respect to the isocenter, is tracked by the system in real time. The nonreflective markers are only used for the isocenter registration. Calibration of the system is performed using a vendor‐supplied calibration apparatus aligned with the room lasers. The spatial accuracy of the system is within 0.8 mm^(8)^ and the mean registration error is approximately 0.3 mm.^(8)^

The kV CBCT system consists of an amorphous silicon (aSi) detector and a kV X‐ray source installed on two retractable arms. The kV X‐ray beam is orthogonal to the Linac's treatment beam. The unit produces diagnostic‐quality X‐rays from 70 to 150 kVp. The active area of the aSi detector is $41\times 41\;{\text{cm}}^{2}$ at the nominal detector‐to‐focal spot distance of 160 cm. The scanning parameters and calibration techniques used for the CBCT have been previously reported.^(5)^ The manufacturer's specification for calibration accuracy is a kV–MV isocenter coincidence of ≤ 1.0 mm.^(^ ^2^ ^,^ ^4^ ^,^ ^5^ ^) (^ We routinely verified the coincidence to within 0.3 mm.) In this study, we acquired the CBCT scans using a $27.6\times 27.6\times 20\;{\text{cm}}^{3}$ field of view (FOV) and a gantry rotation range of 205° over approximately 70 seconds. The CBCT software (v4.0) supports both bony and soft‐tissue fusion, and allows for a user‐defined region of interest (ROI) fusion volume for the registrations.

All three of the IGSPSs' image registrations yield six degree of freedom parameters and display results to submillimeter (mm) and subdegree (°) precision; once the three systems' correlation is defined, the accuracy of the mechanical and imaging QA parameters can be cross‐verified.

The radiation isocenter for our Linac is defined at the CBCT isocenter. The room lasers, optical distance indicator (ODI) at 0° gantry angle, the SonArray system origin, and the VisionRT system origin are defined to match this point.

### C. Description of QA procedures

#### C.1 Mechanical and imaging

The TG 40,^(1)^ TG 142,^(2)^ and TG 104^(4)^ reports recommend QA procedures for mechanical and imaging parameters. The reports recommend that laser localization, ODI at isocenter, and imaging and treatment isocenter coincidence using CBCT be verified on a daily basis.

A modified DQA3 was used to assess the mechanical and dosimetry QA parameters for the Linac and the imaging QA parameters for the three IGSPSs. The adjustment knobs on the bottom of the device were altered to allow it to rotate. The DQA3 was modified by affixing a bite plate with attached reflective markers (Fig. 2). To establish a single QA system which utilizes the modified DAQ3 as the sole QA device for all tests, a reference‐planning CT of the DQA3 was obtained, and the reference isocenter was selected to be at the center of the central ion chamber on the surface of the DQA3 (Fig 2), which was identified using three radiopaque markers. The reference‐planning CT images and reference isocenter were transferred to both the FSA and the CBCT systems.

**Figure 3:**
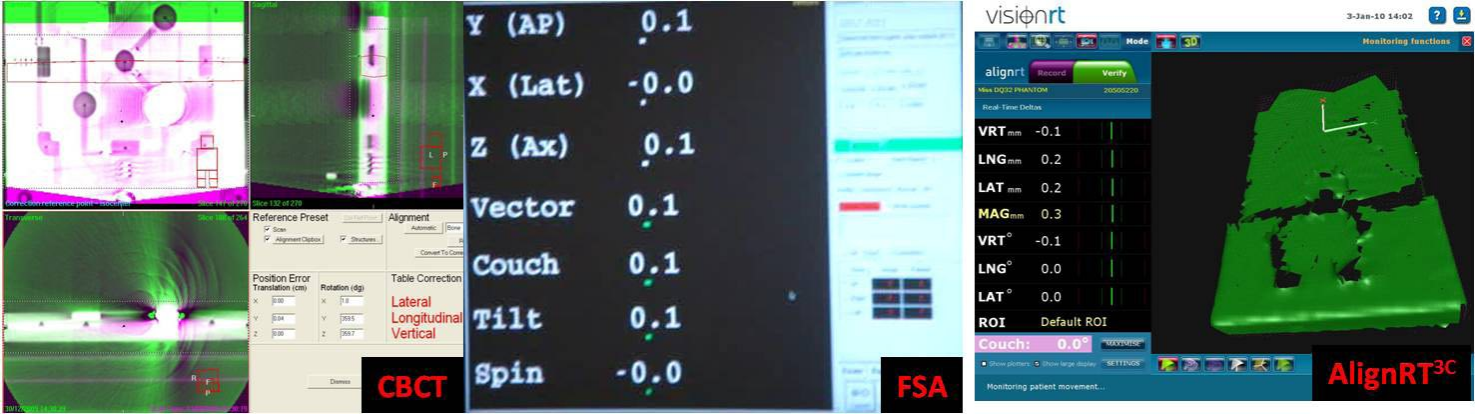
Examples of registration process for the three IGSPSs (CBCT, FSA, and ${\text{AlignRT}}^{3C}$).

The DQA3 was placed on the couch and the FSA system, using the reference isocenter, was used to position the device to ≤ 0.1 mm and ≤ 0.1° (along all three axes) of the Linca radiation isocenter. The device was then localized using the CBCT system to within 0.3 mm and 0.3° of the Linac radiation isocenter. An Align ${\text{RT}}^{3C}$ surface reference image was then captured. Capturing the Align ${\text{RT}}^{3C}$ surface reference image after localizing with the CBCT eliminated the inherent variances between these two systems. Figure 3 displays the image registration screens for the three systems. The laser localization and optical distance indicator (ODI) were then checked.

To obtain statistics on the isocenter position (Linac, CBCT, FSA, and Align ${\text{RT}}^{3C}$), the DQA3 was repositioned on the table ten times, and the above procedure was repeated for each repositioning.

The TG 142^(2)^ and TG 104^(4)^ reports recommend that the couch position indicators (translation and rotation) be verified on a monthly basis. The DQA3 can also be used to evaluate the localization accuracy of the three imaging systems for various couch displacements. For a couch angle of 0°, the couch was shifted to randomly position the DQA3 to two different positions lying within a range of ± 30 mm of the Linac isocenter. At each position, the translational displacement of the DQA3 was determined using the Align ${\text{RT}}^{3C}$, the CBCT, the FSA, and the couch digital readout. A similar evaluation was done using four different couch angles, ± 45° and ± 90°, but with no translation of the couch. For this evaluation, the rotational displacement of the DQA3 was determined at each couch angle using each of the imaging systems. For the four couch angles, the rotational displacements were determined using only the FSA and Align ${\text{RT}}^{3C}$ systems. The CBCT system was not utilized for these tests since it can only be used with the couch at 0°. Both of these repositioning tests were repeated ten times on ten different days over a span of one month. For the translation tests, two random positions were measured each time, for a total of twenty measurements. For the rotation tests, one measurement was taken with each system at each table angle, for a total of ten measurements.

#### C.2 Dosimetry

The daily checks with the DQA3 consisted of X‐ray and electron output constancy, energy verification, beam profile symmetry and flatness checks, radiation field size, and light‐radiation field size coincidence. Measurements were taken with the DQA3 placed at an SSD of 100 cm, with the central chamber located at the CAX.

## III. RESULTS & DISCUSSION

Table 1 gives the isocenter displacement comparisons (means and standard deviations) for the systems for the ten measurements taken with no couch shift. The directional differences along the device's left–right, superior–inferior, and anterior–posterior axes are represented by Δ LAT, Δ LONG, and Δ VERT, respectively (Fig. 1). In a previous CBCT study, a systematic difference between the kV and MV isocenter of 0.3±0.2 mm was observed,^(^ ^4^ ^,^ ^5^ ^)^ and we selected the CBCT as our reference since it is used to define the radiation isocenter. The TG142 report suggests imaging treatment isocenter coincidence tolerances of ≤ 2 mm and ≤ 1 mm for IMRT and SRS, respectively.^(2)^ The isocenter displacement of the CBCT agrees with that of the Linac and with those of the other two imaging systems (Align ${\text{RT}}^{3C}$ and FSA) to within 0.7 mm and 0.6°. Because the Elekta couch is not a six degree of freedom couch, the displacement of the couch is displayed in units of translation and yaw rotation only. The laser aligned to within 0.0 mm of the central radiopaque marker of the DQA3, and was within approximately 0.5 mm (visually) of its two side markers, which was within the suggested TG142 stringent tolerance of 1 mm. The repeated ODI digital readout was within ± 0.5 mm (visually), which was within the TG142 suggested tolerance of 2 mm. The maximum standard deviation of the registration error was 0.4 mm. Many studies^(^ ^4^ ^,^ ^7^ ^,^ ^8^ ^)^ have reported the registration errors for the CBCT, the FSA, and the Align ${\text{RT}}^{3C}$ to be within 0.5 mm of one another.

**Table 1:** Isocenter displacements between the systems (Linac, FSA, and ${\text{AlignRT}}^{3C}$) with respect to the CBCT explored with DailyQA3 tests for 10 repeated measurements. Note that this number will change after recalibration of either system.

	${\text{AlignRT}}^{3C}$ vs. *XVI*		*FSA vs. XVI*		*Linac Mechanical vs. XVI*	
	*Trans (mm)*	*Rot (°)*	*Trans (mm)*	*Rot (°)*	*Trans (mm)*	*Rot (°)*
Δ LAT	0.2±0.4	−0.6±0.1	0.1±0.2	−0.6±0.1	−0.1±0.3
Δ LONG	−0.2±0.2	−0.5±0.1	0.0±0.4	−0.6±0.2	0.4±0.2
Δ VERT	−0.5±0.4	−0.2±0.1	0.5±0.2	0.3±0.2	0.6±0.3
Vectors	0.6±0.3		0.5±0.2		0.7±0.3

Table 2 gives the results of the couch repositioning tests for the 0° couch angle setup. Note that the systematic isocenter differences between the systems were subtracted before doing the comparison. For the mean values, the results show similar differences of 0.44 mm between the systems, which is within the TG 142 suggested tolerance of ≤ 1 mm in repositioning accuracy.

**Table 2:** Comparisons of the positioning variances between the systems (Couch, FSA, and ${\text{AlignRT}}^{3C}$) with respect to the CBCT at couch 0° for 20 displacements within ±30 mm obtained.

	*AlignRT3C vs. XVI*		*FSA vs. XVI*		*Couch vs. XVI*	
	*Trans (mm)*	*Rot (°)*	*Trans (mm)*	*Rot (°)*	*Trans (mm)*	*Rot (°)*
Δ LAT	−0.3±0.5	−0.1±0.2	0.0±0.2	0.0±0.2	0.0±0.5
Δ LONG	−0.2±0.4	0.0±0.1	−0.1±0.4	0.0±0.2	0.1±0.5
Δ VERT	0.2±0.5	0.1±0.3	−0.2±0.5	−0.1±0.3	−0.3±0.7
Vectors	0.4±0.5		0.2±0.4		0.3±0.7

Since the system correlation was defined at a couch angle of 0°, the rotational differences between the systems were 0.1° ± 0.3°.

In addition to our daily QA feasibility study, we can also use both the FSA and the ${\text{AlignRT}}^{3C}$ with the DQA3 to check the couch rotational accuracy. Table 3 gives the results for the tests performed for the four non‐zero couch angles (45°, 90°, 135°, and 270°). The measured rotational offsets in the vertical direction (pitch) were within −0.3° ± 0.1° for the Align ${\text{RT}}^{3C}$ and 0.0° ± 0.1° for the FSA, which are within the TG142 recommendation of 1° for the table angle for IMRT.^(2)^ The rotational variances in the longitudinal (yaw) and lateral (roll) directions were all less than 0.5° for both systems.

**Table 3:** Comparisons of the accuracy of the couch angles between the systems (Couch, FSA, and ${\text{AlignRT}}^{3C}$) at four couch angles (45°, 90°, 135°, and 270°) with 10 repeated measurements.

	${\text{AlignRT}}^{3C}$	*Couch* $45^{0}$ *FSA*	*Couch*	*Couch* $135^{0}$	${\text{AlignRT}}^{3C}$ FSA	*Couch*
Δ LAT	0.0±0.1	0.5±0.1		−0.2±0.2	−0.3±0.1	
Δ LONG	0.3±0.2	0.5±0.4		0.0±0.1	0.3±0.1	
Δ VERT	−0.3±0.1	0.0±0.0	0.0	0.0±0.2	0.0±0.0	0.0
	${\text{AlignRT}}^{3C}$ *FSA*	*Couch* $90^{0}$	*Couch*	${\text{AlignRT}}^{3C}$	*Couch* $270^{0}$ *FSA*	*Couch*
Δ LAT	0.0±0.2	0.5±0.1		−0.2±0.2	−0.1±0.2	
Δ LONG	0.3±0.1	0.5±0.4		0.2±0.1	0.1±0.2	
Δ VERT	−0.2±0.1	0.0±0.1	0.0	0.0±0.1	0.0±0.1	0.0

**Figure 4:**
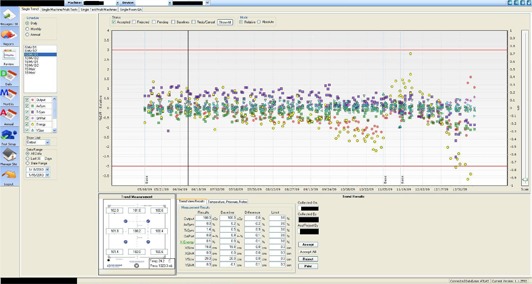
Daily QA3 software provides a graphical representation of data for each template.

Table 4 tabulates the dosimetric results for the daily QA procedures for the Linac including output, energy, symmetry and flatness, field size, and light‐radiation coincidence. The DQA3 software allows us to record automatically each measurement and to view long‐term variation (Fig. 4). Users have the option to choose which data to view for a specific time period over which the data were taken. A single measurement can be examined by clicking on a data point for a particular day. For over eight months, we were able to verify that the output remained within 2%, the photon and electron energy remained within 2%, and the symmetry and flatness remained within 1%. The data show that the field size remained within 1 mm with approximately 1 mm standard deviation, and that the light‐radiation coincidence remained within 1 mm. In summary, the readings are within the AAPM recommendations.^(^ ^1^ ^,^ ^2^ ^)^

**Table 4:** Dosimetric results following recommended daily QA procedures of the linac over eight months.

	*Output (%)*	*Energy (%)*	*Sym. (%)*	*Flat. (%)*	*F.S. (mm)*	*Light and Radiation F.S. Coincidences (mm)*
*Tolerances* ^(^ ^1^ ^,^ ^2^ ^)^	*3% (Daily) 2% (Monthly)*	*2%/2 mm*	±1%	*1.0%*	*2 mm (IMRT) 1 mm (SRS)*	*2 mm/1%*
6 MV	0.0±0.8	0.0±2.6	0.0±0.3	0.0±0.3	0.0±0.0	0.0±0.1
10 MV	0.0±0.7	0.0±1.9	0.0±0.5	0.0±0.2	0.0±0.0	0.0±0.1
18 MV	0.0±0.4	0.0±1.3	0.0±0.5	0.0±0.1	0.0±0.0	0.0±0.1
6 MeV	0.0±0.5	0.0±0.4	0.0±0.4	0.0±0.1	0.0±0.1	0.0±0.1
8 MeV	0.0±0.5	0.0±0.2	0.0±0.5	0.0±0.1	0.0±0.1	0.0±0.0
10 MeV	0.0±0.5	0.0±0.4	0.0±0.6	0.0±0.1	0.0±0.1	0.0±0.0
12 MeV	0.0±0.5	0.0±0.6	0.0±0.3	0.0±0.1	0.0±0.1	0.0±0.0
15 MeV	0.0±0.5	0.0±0.3	0.0±1.0	0.0±0.1	0.0±0.1	0.0±0.0
18 MeV	0.0±0.7	0.0±0.6	0.0±0.6	0.0±0.2	0.0±0.1	0.0±0.0
20 MeV	0.0±0.5	0.0±0.4	0.0±0.5	0.0±0.1	0.0±0.1	0.0±0.0

Abbreviations: Sym.=symmetric of dose profile; Flat.=flatness of dose profile; F.S.=field Size; IMRT=intensity‐modulated radiotherapy; SRS=stereotactic radiosurgery

To perform the daily QA CBCT radiation isocenter check, a S10 collimator cassette is used. The nominal irradiated length on this cassette is approximately 13 cm, and it was chosen to minimize radiation exposure to the electronics of the DQA3 device. After more than a year of utilizing the DQA3 for comprehensive QA, no evidence of radiation damage was seen. For CBCT, the image fusion of the DQA3 gave consistent results, which is due to the CT image of the complex chamber structures of the device.

The room lasers are used to align the FSA calibration device. The estimated calibration uncertainty for the FSA should be within 0.5 mm of the defined radiation isocenter. There is no difference between the ${\text{AlignRT}}^{3C}$ and CBCT systems since the ${\text{AlignRT}}^{3C}$ reference image is captured after the DQA3 is aligned using the CBCT.

## IV. CONCLUSIONS

The key purpose of our study was to streamline the Linac daily QA by combining the daily dosimetry QA with all of the daily IGRT QA. The QA system and procedure are applicable to any IGRT system. The total time to finish all of the daily QA was less than 15 minutes including device setup (< 3 minutes), verification of the Linac isocenter with the three IGSPSs (< 5 minutes), and measurement of the Linac dosimetry (three photon energies and two electron energies < 4 minutes). We also designed a template that lists the QA parameters for the therapists to aid in streamlining the process. In addition, as part of our monthly QA, couch positioning/ repositioning measurements for two couch translational positions (couch = 0°) and two couch angles (no couch translation) took less than 10 minutes. In conclusion, the DQA3 can be used efficiently and reliably for daily LINAC and IGSPS QA.
